# Antibiotics in Dentistry: A Narrative Review of the Evidence beyond the Myth

**DOI:** 10.3390/ijerph20116025

**Published:** 2023-06-01

**Authors:** Maria Contaldo, Francesco D’Ambrosio, Giuseppe A. Ferraro, Dario Di Stasio, Maria Pia Di Palo, Rosario Serpico, Michele Simeone

**Affiliations:** 1 Multidisciplinary Department of Medical-Surgical and Odontostomatological Specialities, University of Campania “Luigi Vanvitelli”, 80138 Naples, Italy; giuseppe.ferraro@unicampania.it (G.A.F.); dario.distasio@unicampania.it (D.D.S.); rosario.serpico@unicampania.it (R.S.); 2 Department of Medicine, Surgery and Dentistry, University of Salerno, 84081 Salerno, Italy; mariapia140497@gmail.com; 3 Department of Neuroscience, Reproductive Science and Dentistry, University of Naples Federico II, 80138 Naples, Italy

**Keywords:** antibiotic, antibiotics, antimicrobial stewardship, drug resistance, dentistry, dentists, dental health services

## Abstract

Antibiotics have undoubtedly revolutionized medicine and the health and survival of patients with life-threatening infections, being nonetheless free from potential adverse effects, and the risk of intestinal dysbiosis, antimicrobial resistance, and the resulting consequences for the patient’s health and the public purse. The present study narratively reviewed the epidemiological data on worldwide antibiotic consumption and administration in dental practice, patients’ adherence to prescriptions, the antimicrobial resistance phenomenon in dentistry, and the evidence supporting and recommending appropriate antibiotic use in dental care. Eligible systematic reviews and original studies in humans published in the English language from January 2000 to 26 January 2023 were considered. A total of 78 studies, 47 on the epidemiology of antibiotic use and prescription in dentistry, 6 on antibiotic therapy in dentistry, 12 on antibiotic prophylaxis in dentistry, 0 on adherence of dental patients to antibiotic prescription, and 13 on antimicrobial resistance in dentistry, were presently considered. Retrieved evidence revealed that antibiotics are frequently overused and misused in dental practice, dental patients frequently do not adhere to prescriptions, and antimicrobial resistance in dentistry is a still rising phenomenon also secondary to improper oral antiseptics use. The present findings highlighted the need to establish more evidence-based and accurate antibiotic prescriptions to sensitize dentists and dental patients to minimize and rationalize the use of antibiotics only when it is indicated and necessary, improve patients’ adherence, and enhance knowledge and awareness of the antimicrobial resistance in dentistry.

## 1. Introduction

The development and use of antibiotics were one of the most important discoveries of the 20th century, revolutionizing the treatment of infectious diseases, thus saving millions of lives [1] and enabling critical advances in many medical fields [2]. An antibiotic is defined as “a substance produced by microorganisms that can act on other microorganisms (or living cells) by inhibiting their growth or destroying them (antibiotic action)”. Indeed, the term, coming from the Greek and meaning “against life”, is thus used to refer to drugs that can inhibit or slow down the multiplication of bacteria [3,4], either by inhibiting one or more specific metabolic pathways essential to the bacterium or by acting on a specific target of the bacterial cell [5,6].

Most antibiotics used in medicine today are compounds produced naturally by bacteria or fungi, despite how they can also be purely synthetic or semisynthetic compounds, being thus referred to as chemotherapeutic agents [7]. Among them, the β-lactams, macrolides, lincosamides, nitroimidazoles, and tetracyclines are certainly the most commonly used classes of antibiotics and are best suited for dental problems [8,9,10,11,12]. Antibiotics are generally prescribed in dentistry for the treatment of odontogenic and non-odontogenic acute and chronic infections, for the prophylaxis of focal infections in high-risk patients (those suffering from systemic diseases such as endocarditis or congenital heart disease), and of focal, systemic, and surgical site infections in patients requiring dental treatment or oral surgery [13]. Antibiotic prescribing in dentistry, whether for prophylactic or therapeutic purposes, accounts for approximately 10% of antibiotic prescriptions worldwide [13,14], and is not always considered appropriate, leading to excessive or incorrect antibiotic use in dental practice [13,14]. In turn, dental patients may not correctly adhere to prescribed antimicrobial treatment, exacerbating the overuse and misuse of antibiotics in dentistry [15].

Indeed, the potential benefit of antibiotic administration must be weighed against the risk of side effects, such as severe allergic reactions and anaphylaxis, infections caused by the bacterium *Clostridioides difficile*, and antibiotic-induced colitis [14]. Specifically, in the use of broad-spectrum antibiotics, the direct action even against saprophytic bacterial species regarded as “good” for the body has several consequences for human health that should not be underestimated and that require greater awareness and rationalization of their use, both on the part of the doctor or dentist and the patient. Indeed, taking live cultures or probiotics during antibiotic therapy can alleviate some of the problems associated with taking these drugs and restore good bacteria. In fact, probiotics maintain the balance of the intestinal flora and restore the conditions that existed before the dysbiosis caused by antibiotics [16].

In light of these considerations, the present study narratively reviewed the epidemiological data on global antibiotic consumption and antibiotic administration in dental practice, prescription adherence of dental patients, and the phenomenon of antimicrobial resistance in dentistry, as well as the evidence supporting and recommending appropriate antibiotic use in dental care.

## 2. Materials and Methods

The present narrative review focused the research questions on the current prevalence of antibiotic administration in dental practice for therapeutic and prophylactic purposes, reported indications for antibiotic prescriptions, and evidence concerning dental patients’ adherence to the therapy, antimicrobial resistance phenomenon, and antibiotic stewardship in dentistry, as well as the evidence supporting and recommending appropriate antibiotic use in dental care.

### 2.1. Search Strategy and Eligibility Criteria

An electronic search of the PubMed/MEDLINE, Web of Science, and Scopus databases was conducted through 26 January 2023 to find pertinent records. The present study had the systematic reviews and original human studies published in English from January 2000 to 26 January 2023 as the inclusion criteria, with the epidemiology of antibiotic use and prescription in dentistry; or antibiotic therapy in dentistry; or antibiotic prophylaxis in dentistry; or adherence of dental patients to antibiotic prescription; or antimicrobial resistance in dentistry as the main topics. Exclusion criteria were as follows: narrative and scoping reviews, commentaries, short communications, in vitro, preclinical and animal studies, non-English language articles, and being published before January 2000.

The following filters were used for the electronic search of the PubMed database: Classical Article, Clinical Study, Clinical Trial, Comparative Study, Controlled Clinical Trial, Evaluation Study, Meta-Analysis, Multicenter Study, Observational Study, Randomized Controlled Trial, Systematic Review, Validation Study, English language, and published articles from 1 January 2000 to 26 January 2023. The following filters were used for the electronic search of the Web of Science database: English language and published articles from 1 January 2000 to 26 January 2023. The following filters were used for the electronic search of the Scopus database: Article, Review, English language, and published articles from 2000 to 2023.

The following keywords were used:

- Antibiotic OR Antibiotics

AND

- Stewardship OR Administration OR Prescription OR Use OR Adherence OR Compliance OR Resistance

AND

- Bacterial resistance OR Antimicrobial resistance

AND

- Dentist OR Dentistry.

Relevant references were collected, and related citations were managed using Mendeley Reference Manager software. Study selection was independently conducted by three reviewers (M.C., F.D., and M.P.D.P.).

### 2.2. Data Extraction and Collection

Three independent reviewers (M.C., F.D., and M.P.D.P.) extracted the data using a standardized form for data extraction. In case of any disagreements, the reviewers resolved them through discussion, and if necessary, a third author (R.S.) was consulted to reach a consensus.

The author’s name, the year and journal of publication, the reference number, and the title of the study were collected for each record.

The studies were categorized into five main topics: epidemiology of antibiotic use and prescription in dentistry; antibiotic therapy in dentistry; antibiotic prophylaxis in dentistry; adherence of dental patients to antibiotic prescription; antimicrobial resistance in dentistry.

## 3. Results

### 3.1. Study Selection

A total of 287 records were found from the electronic search, 50 from PubMed/MEDLINE, 123 from Scopus, and 114 from Web of Science databases; 93 duplicate records were removed before the screening. The remaining 194 titles and abstracts were screened, and 51 were excluded. The full texts of the remaining 143 reports assessed for eligibility were evaluated. Additionally, 65 articles were excluded because 40 records were narrative reviews, 17 involved only dental students, 5 did not report the use of antibiotics in dental practice, and in 3 articles, data concerning dentistry were not discernible.

**Figure 1:**
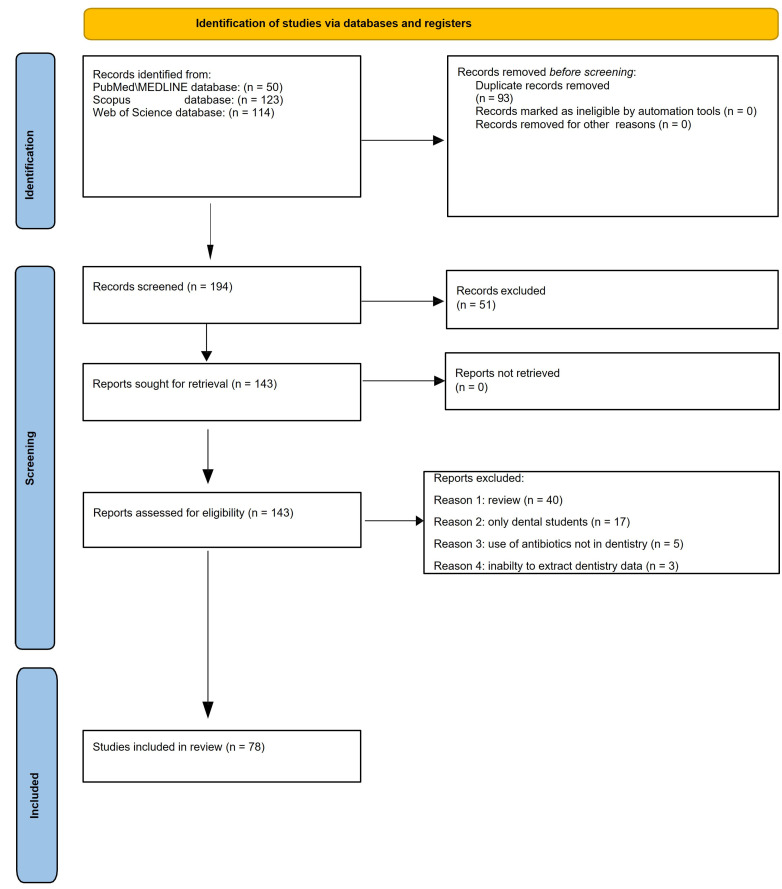
PRISMA 2020 flow diagram for identification of studies via databases.

Finally, 78 studies were included (Figure 1).

An additional electronic search was performed on the Google Scholar database to obtain additional records for discussion of the results.

### 3.2. Study Characteristics

The 78 studies included were categorized into five main topics: 47 on the epidemiology of antibiotic use and prescription in dentistry, 6 on antibiotic therapy in dentistry, 12 on antibiotic prophylaxis in dentistry, 0 on adherence of dental patients to antibiotic prescription, and 13 on antimicrobial resistance in dentistry.

In the present review, of the 78 included studies, 48 were cross-sectional studies, 9 were systematic reviews, 8 were retrospective studies, 5 were clinical trials, 4 were systematic reviews with meta-analysis, 2 were randomized control trials, 1 was a comparative study, and 1 was a qualitative study.

### 3.3. Studies Reporting Epidemiology of Antibiotic Consumption and Prescription in Dentistry

In the present review, of the 47 studies reporting the epidemiology of antibiotic consumption and prescription in dentistry, 38 were cross-sectional studies, 4 were retrospective studies, 4 were systematic reviews, and 1 was a randomized control trial.

Table 1 presents the author’s name, the year and journal of publication, the reference number, the title and type of study, and the main conclusion(s) about the epidemiology of antibiotic consumption and prescription in dentistry.

The findings of the various studies about the epidemiology of antibiotic consumption and prescription indicated a lack of adherence to professional guidelines in prescribing antibiotics in dentistry. Only a small percentage of dentists refer to published guidelines for proper antibiotic prescribing, and an even smaller percentage were aware of national action plans on antibiotic resistance [17,18,20,21,28,29,54]. There was still also a lack of knowledge and uniformity in antibiotic prescriptions among dentists in different countries, including Saudi Arabia [18], Pakistan [17], Kuwait [52], Ghana [54], Italy [5], United Kingdom [25], Croatia [30], India [23], Lebanon [39], Northern Ireland [40], and Israel [57]. The survey results highlighted a divergence in antibiotic prescribing practices also among different dental specialists [18,19,38].

Antibiotic overprescription, in particular of amoxicillin or other broad-spectrum antibiotics, was prevalent in cases where surgical treatment was the preferred choice, irreversible pulpitis, necrotic pulp, acute apical periodontitis, and endodontic emergencies [20,22,31,42,53].

The COVID-19 pandemic significantly impacted antibiotic prescribing, with a substantial increase in prescriptions during and after the pandemic [27].

Factors influencing antibiotic prescriptions in dentistry were identified as clinical context-related (dentists working in different sectors exhibited variations in antibiotic prescribing practices [41,48]), patient-related [25,26,52,58], social–political context-related [61], and clinician-related (dentists with fewer years of practice tend to exhibit lower rates of antibiotic prescription abuse [60]).

Educational interventions, postgraduate courses, and training programs have positively improved appropriate antibiotic prescribing in dentistry [22,36]. Dentists who received additional education on antibiotic use demonstrated greater knowledge and better prescribing practices [22,36]. Many dentists also expressed a desire to receive more information on the proper use of antibiotics [21].

### 3.4. Studies Reporting Antibiotic Therapy in Dentistry

In the present review, of the six studies reporting antibiotic therapy in dentistry, four were cross-sectional studies, one was a qualitative study, and one was a systematic review with meta-analysis.

Table 2 presents the author’s name, the year and journal of publication, the reference number, the title and type of study, and the main conclusion(s) about antibiotic therapy in dentistry.

The findings of the studies about antibiotic therapy in dentistry highlighted the need for better adherence to guidelines and more appropriate prescribing patterns among dentists [64,68].

In periodontal and implant practice, there was significant heterogeneity in the prescription, duration, and initiation of antibiotics across various therapeutic treatments. These treatments include acute and chronic periodontitis, sinus lifts or crest augmentation surgery, and immediate or delayed implant placement [64]. Nonetheless, despite the overprescriptions of systemic antibiotics in non-surgical periodontitis treatments, no statistically significant evidence was found regarding their long-term efficacy [66]. Additionally, there is no defined superiority of one antibiotic over others [66].

The prescription of antibiotics by dentists for the treatment of inflammatory endodontic diseases was also inappropriate, indicating a lack of adherence to recommended prescribing practices [67].

The study revealed the overuse of antibiotics and analgesics, particularly in the postoperative phase, by general dentists rather than specialists, for pain control and treatment of infections, even in children and adolescents [65].

The general practitioner often considered antibiotics as the first-line treatment for the immediate management of acute dental problems, and patients themselves often requested antibiotics [63].

### 3.5. Studies Reporting Antibiotic Prophylaxis in Dentistry

In the present review, of the 12 studies reporting antibiotic prophylaxis in dentistry, 4 were cross-sectional studies, 4 were systematic reviews, 2 were retrospective studies, 1 was a systematic review with meta-analysis, and 1 was a randomized control trial.

Table 3 presents the author’s name, the year and journal of publication, the reference number, the title and type of study, and the main conclusion(s) about antibiotic prophylaxis in dentistry.

The findings of the studies about antibiotic prophylaxis showed the heterogeneity in the choices made by dentists for different procedures [72]. Responses were more heterogeneous for the need or not of antibiotic prophylaxis for adult extractions with comorbidities, complex or multiple extractions, drainage abscesses, and implant placement, while more appropriate choices were reported for deciduous tooth extractions and simple extractions in healthy adult subjects [70,72]. Antibiotics were commonly prescribed to prevent infection at the surgical site or to reduce bacteremia [70,74]. Dosages also varied significantly [70].

The choices made by dentists regarding antibiotic prescriptions for preventing infections in post-extraction surgical sites were very heterogeneous [71]. The use of antibiotics prophylactically for third-molar extractions in healthy patients had limited supporting evidence [71,79]. Oral antibiotics administered during routine surgical extractions of third teeth in the absence of inflammation did not show a reduction in post-operative complications, greater benefit in patient-related outcome measures, or a higher incidence of adverse events [79].

Amoxicillin, with or without clavulanic acid, was found to be the most commonly prescribed antibiotic, at varying dosages and durations, for preventing infectious complications following the extraction of third molars [78]. Compared to using third-generation cephalosporins, antibiotic prophylaxis with amoxicillin prior to extraction of impacted third molars was associated with a lower incidence of surgical site infections [76,77]. In cases of penicillin allergy, alternative antibiotics such as azithromycin, clarithromycin, or metronidazole were recommended, while clindamycin was advised against if possible.

The use of antibiotics prophylactically for implant placement had limited supporting evidence. There was high variability in the pre-and post-operative prescription regimens of antibiotics in dental implant practice [80]. Prophylactic treatment with 2–3 g of amoxicillin one hour before immediate implant placement and continued for 5–7 days at a dosage of 500 mg every 8 h was found to be effective in reducing the rate of early implant failure [73].

The prophylactic administration of 2/3 g of amoxicillin one hour before bone augmentation procedures in oral implantology was found to reduce the rate of early implant failure and the infection risk of grafted bone particles [75].

### 3.6. Studies Reporting Dental Patients’ Adherence to Antibiotic Prescription

In the present review, no study reporting dental patients’ adherence to antibiotic prescriptions was found. Table 4 presents the author’s name, the year and journal of publication, the reference number, the title and type of study, and the main conclusion(s) about dental patients’ adherence to antibiotic prescription.

### 3.7. Studies Reporting Antimicrobial Resistance in Dentistry

In the present review, of the 13 studies reporting antibiotic antimicrobial resistance in dentistry, 5 were clinical trials, 2 were systematic reviews with meta-analysis, 2 were cross-sectional studies, 2 were retrospective studies, 1 was a comparative study, and 1 was a systematic review.

Table 5 presents the author’s name, the year and journal of publication, the reference number, the title and type of study, and the main conclusion(s) about antimicrobial resistance in dentistry.

The findings of the studies on antimicrobial resistance highlighted the spread of antibiotic resistance [83,84], in particular among the common pathogens that were implicated in the pathogenesis of periodontal [92] and endodontic diseases [86,88,93].

Ampicillin, tetracyclines, and metronidazole showed the highest resistance rates, while azithromycin and ciprofloxacin had the lowest [81,85,86,87,91].

The resistance increased with repeated cycles of antibiotic administration for the selection of microorganisms [90]. Frequent use of chlorhexidine also resulted in increased resistance rates of microorganisms to chlorhexidine itself and other antibiotics [89].

## 4. Discussion

### 4.1. Epidemiology of Antibiotic Consumption and Prescription in Dentistry

Antibiotic consumption is steadily increasing worldwide, with penicillins being the most used class of broad-spectrum antibiotics in dentistry [94]. In Australia, for example, 11 of the 20 drugs most prescribed by dentists are antibiotics, including amoxicillin, an antibiotic in the penicillin family [95]. Similarly, in the United States of America, 10% of all antibiotic prescriptions are written by dentists [96].

Unfortunately, a British study found that 80% of antibiotics used to treat acute dental diseases were unnecessary [97]. Another study conducted in the United States found that antibiotics were used inappropriately for prophylaxis in 80% of cases [98]. Therefore, the administration of antibiotics among physicians and dentists [94] has steadily increased, leading to misuse and overuse of antibiotics that has unfortunately contributed to an increase in bacterial resistance to antimicrobial agents [37,95], leading in turn to higher mortality rates, longer hospital stays, and reduced protection of patients from infectious diseases.

Antimicrobial resistance is a public health problem that requires a global solution and, if not controlled, will have high human and economic costs. During the COVID-19 pandemic, the phenomenon of antimicrobial resistance continued to accelerate dramatically [27], and its full consequences will likely not be seen for years to come [99]. In fact, it is estimated that by 2050, 10 million deaths annually will be secondary to multidrug-resistant microorganisms, costing many billions of dollars [100].

The ineffectiveness of interventions implemented worldwide is evidenced by the fact that between 2000 and 2015, antibiotic consumption in 76 countries, expressed in defined daily doses (DDDs), increased by 65% (21.1–34.8 billion DDDs), and the rate of antibiotic consumption increased by 39% (11.3–15.7 DDDs per 1000 population per day) [94].

In response to this growing global threat, the World Health Organisation launched a Global Action Plan on Antimicrobial Resistance in 2015 and published the Global Action Plan on Antimicrobial Resistance. Therefore, knowing the type and dosage of recommended antibiotics and the measures to combat antimicrobial resistance in dentistry is of utmost importance.

### 4.2. Antibiotic Therapy in Dentistry: Current Measures

Odontogenic infections are a group of diseases originating in the dental hard tissues or periodontium from microorganisms constituting dental and gingival biofilm. If not promptly intercepted and treated, they can lead to severe local or systemic complications. Odontogenic infections may derive from dental caries, trauma, pulpitis, periapical periodontitis, endoperiodontal lesions, pericoronitis, and periodontal disease. The most common cause of infection is periapical periodontitis, which in most cases is due to destructive caries, with the most affected teeth being the lower molars [101].

Non-odontogenic infections include pyogenic infections of the face and neck region, oral mucosal infections, oropharyngeal candida, parotitis, and sialadenitis [102,103,104].

Despite the high incidence of odontogenic infections, there are no specific criteria for prescribing antibiotics in this area. A high percentage of cases of dental pain are due to pulp infections, which require operative intervention by the dentist rather than antibiotic treatment [13].

Clinical situations requiring antibiotic therapy on an empiric basis are limited to and include oral infections associated with signs of systemic spread of infection [17,65], such as elevated body temperature, lymphadenopathy, and lockjaw [13]. Coherently, antibiotics are not indicated for all odontogenic infections and should not be used in place of eliminating the cause of infection [72].

According to the pathophysiology of pulp disease, blood circulation in the root canals is reduced; therefore, antibiotics cannot reach the pulp and eliminate the pathogens [41,57]. Only endodontic intervention can relieve the symptoms and eliminate the infection [55]. Nevertheless, dentists around the world continue to prescribe antibiotics for localized infections without systemic involvement [57,67,105]. Even for periodontitis and peri-implantitis, systemically delivered antibiotics are not indicated [106,107,108].

Of note, a systemic antibiotic prescription is recommended for necrotizing gingivitis and periodontitis [106,107,108], stage III-grade C and incisor molar periodontitis (previously referred to as aggressive localized periodontitis, acute periapical abscess, cellulitis, and pericoronitis).

Oral antibiotics effective for odontogenic infections include penicillin, clindamycin, erythromycin, cefadroxil, metronidazole, and tetracyclines. The type of antibiotics or their combinations and dosage depend on the severity of the infection and the predominant bacterial species [13]. However, the most prescribed antibiotic in dentistry is amoxicillin, followed by amoxicillin + clavulanic acid [32,62,105].

#### Antibiotic Therapy in Dentistry: Guidelines for More Judicious Use of Antibiotic Therapy in Dentistry

**Figure 2:**
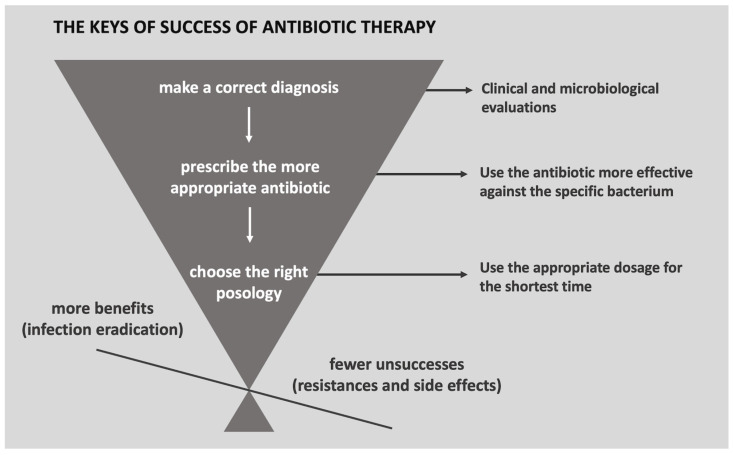
The success of antibiotic therapy requires a correct preliminary diagnosis of the bacterial infection, when possible, supported by microbiological assessments to identify the species and strain involved. Then, the more appropriate antibiotic must be chosen based on the support of an antibiogram, when feasible, or the current edge. Lastly, the posology must consider the shorter time and the appropriate dosage for each case.

Guidelines for the judicious use of antibiotics have been published, suggesting that an accurate diagnosis should be obtained before prescribing a drug and that the specific antibiotic for the bacterium causing the infection should be used, rather than broad-spectrum antibiotics. The duration of treatment should be determined by the shortest amount of time required to eliminate all bacteria causing the infection, as shorter antibiotic cycles reduce the time the bacterium is exposed to the antibiotic and lower the rate at which the pathogen develops resistance, as well as reduce side effects and costs [105]. Additionally, the appropriate dosage should be used for the shortest possible time [109]. Patients should be advised to complete antibiotic treatment, even if symptomatology improves, to prevent the emergence of resistance [105] (Figure 2).

### 4.3. Antibiotic Prophylaxis in Dentistry

Prophylaxis of local infections includes the preoperative, intraoperative, and postoperative administration of antibiotics to prevent the multiplication and spread of bacteria at the level of the surgical lesion [69,79]. Various surgical procedures are performed under antibiotic prophylaxis, including extraction of impacted third molars, orthognathic surgery, implant surgery, and periapical surgery. There is little evidence that antibiotics are effective in preventing infections at surgical sites in the oral cavity, indicating that preoperative parenteral prophylaxis is unwarranted for third-molar extraction in healthy patients [13].

The use of antibiotics for systemic prophylaxis is a common practice [42,66,97]. However, although oral microorganisms can spread from the oral cavity after invasive procedures and colonize distant tissues, there is no strong evidence that this occurs. Therefore, it is controversial when and for which systemic disease prophylaxis is necessary [70]. However, immunocompromised patients are more susceptible to bacteremia, which can rapidly progress to sepsis. Similarly, subjects with uncontrolled diabetes are also considered at higher risk of infection following dental and periodontal procedures due to their reduced immune ability. In both cases, antibiotic care is mandatory for invasive dental procedures [13].

In the case of bacterial endocarditis, which is an inflammatory, proliferative, and exudative condition of the endocardium caused by a bacterial infection involving the heart valves, the absolute risk after dental procedures, even in high-risk patients, is considered very low [85,110].

#### Antibiotic Prophylaxis Prescription in Dentistry

The two principal indications for antibiotic prophylaxis in dentistry are the prevention of bacterial endocarditis and the prevention of surgical site infection (SSI) [111,112,113,114].

For bacterial endocarditis prevention, the British Society for Antimicrobial Chemotherapy and American Heart Association guidelines recommend antibiotic prophylaxis only for high-risk patients [110,112,113]. High-risk patients include those with artificial heart valves, a history of endocarditis, and congenital heart disease causing cyanosis [13]. These recommendations were based on the following evidence: no valid association was found between dental and non-dental procedures and the development of bacterial endocarditis; daily tooth brushing poses an increased risk for bacterial endocarditis due to the exposure of oral flora to bacteremia; the clinical efficacy of antibiotic prophylaxis has not been proven; antibiotic prophylaxis against bacterial endocarditis during dental procedures could lead to a higher number of deaths due to fatal anaphylaxis [110]; proper control of bacterial load and contamination in the oral cavity by eliminating infectious foci and dental biofilm and good periodontal health, along with atraumatic surgical techniques, are the most critical factors affecting the success rate of procedures, rather than the administration of antibiotics [15,115,116]; and antibiotics must be considered a pharmacological adjunct that cannot cover or replace medical interventions [117].

The National Institute for Health and Care Excellence (NICE) recommended antibiotic prophylaxis for high-risk subjects, such as those with prior endocarditis and heart valve replacement, undergoing high-risk dental procedures, including tooth extractions, scaling, and root planing, or undergoing periodontal surgery, by administering amoxicillin 3 g (or clindamycin 600 mg) orally 1 h before the procedure [114].

For the prevention of surgical site infection, in addition to sterile surgical technique, proper perioperative administration and antibiotic selection is considered necessary for most surgical procedures performed on the mucous membranes of the respiratory, gastrointestinal, and urinary tract and in the presence of an active infection. However, antibiotic prophylaxis is not required for most dentoalveolar procedures [111,118,119].

The use of prophylactic antibiotics in tooth extractions is a topic that has been extensively studied, but the findings are mostly limited to third-molar extractions [76,78]. In these procedures, antibiotics should depend on the depth of impaction, the need for osteotomy, trauma to the surrounding tissues, and postoperative inflammation. Although there is no clear evidence on the optimal prophylaxis timing, preoperative antibiotics were found able to reduce dry socket and wound infection rates, and to reduce the risk of infection by 60–70% after the third-molar extraction. However, the incidence of postoperative infections is estimated to be less than 1%, so the potential risks and adverse effects of antibiotics and the risk of antibiotic resistance must be considered [120,121,122,123].

For dental implant placement, there is moderate evidence that preoperative prophylactic antibiotics reduce the risk of implant loss by up to 2%, although likely unnecessary in low-risk patients undergoing single implant surgery. When prophylactic antibiotic administration is warranted, preoperative dosing is recommended. A preoperative dose of 2 g of amoxicillin has been shown to produce the same reduction in implant failure as a dose of 1 g with an additional two days of postoperative treatment [124,125].

Most studies support preoperative prophylactic antibiotic administration in oral and maxillofacial surgery in patients with serious underlying diseases or immunocompromised patients, such as those undergoing radiotherapy or chemotherapy [74,80,126]. However, using antibiotics beyond the first 24 h is of little value in head and neck surgery alone, as SSIs occur in less than 1% of cases, no significant reduction in SSIs in clean head and neck surgery with antibiotic prophylaxis could be observed, and an effective duration could not be determined [118,127]. Further research is needed to find an effective alternative to clindamycin in penicillin-sensitive patients [111].

### 4.4. Dental Patients’ Adherence to Antibiotic Prescription

In combating the phenomenon of antimicrobial resistance, one of the most important measures is to ensure patient adherence to therapy.

Adherence to therapy can be understood as the extent to which a person’s behavior conforms to recommendations agreed upon by a healthcare provider. Patient adherence to antibiotic therapy is paramount in achieving therapeutic success and reducing the development of resistant bacterial strains. As a counterpart, nonadherence to drug therapy is a complex problem influenced by several non-modifiable and modifiable factors [5].

The influence of non-modifiable factors such as age and gender on patients’ adherence to antibiotic prescription is still uncertain, the available evidence is still insufficient and often contradictory, and it is currently difficult to determine whether they influence adherence [128].

Conversely, modifiable factors, on the other hand, significantly influence adherence and can be improved by targeted interventions.

Knowing how to communicate with one’s patient is vital to improve adherence [58], and it is essential that the patient understands the recommendations and the prescribed therapy and that the dentist knows how to communicate them. Indeed, there is a risk that the instructions dentists give to their patients will be misunderstood, forgotten, or even ignored [129].

Cognitive and educational interventions can improve adherence by increasing the patient’s awareness of his condition and the purpose of the prescribed therapy and motivating him to follow the dentist’s instructions [130].

Moreover, combining medication administration with daily activities and interventions that affect patient behavior correlates with improved adherence [130].

Furthermore, interventions based on electronically monitored adherence feedback have been shown to be effective, with an average increase in adherence of almost 20% [130].

Simplification of drug therapy is desirable whenever possible, as a complex drug regimen is associated with lower adherence. In fact, treatment adherence is inversely related to the number of drug doses administered daily, being highest at one or two doses per day [131].

The adherence measurement also has a significant positive impact when performed regularly [132]. There are several methods to measure adherence, each with its advantages and disadvantages:

- Direct methods are generally not applicable to dental patients and measuring drug levels in body fluids, such as blood or urine, or measuring biomarkers.
- Indirect methods include electronic adherence monitoring devices, pill counting, pharmacy refill rates, and self-report [6,131].

Direct measurement involves medications or biomarkers at regular or irregular intervals. It is an accurate and objective method that detects the patient’s medication use. However, it is a more invasive and costly procedure than other methods and does not allow an understanding of the causes of non-adherence. In addition, there is a risk of bias if the patient takes the medication just before the measurement [133].

The main indirect methods of measuring adherence are electronic monitoring, pill counting, pharmacy refill rates, and self-report.

### 4.5. Antimicrobial Resistance in Dentistry

The mechanisms that bacteria use to become resistant to antibiotics can be divided into four different classes [134]: determining modifications of the antibiotic molecule; prevention of reaching the target site; modification or evasion of the target site; and resistance due to adaptation processes of the cell.

Several factors are involved in the development of antimicrobial resistance, mainly distinguishable into intrinsic factors due to natural bacterial resistance to one or more antibiotics, and acquired factors occurring when a population ordinarily sensitive to a given antibiotic becomes resistant to a given antibiotic thanks to the mutation of one or more genes (endogenous acquisition), or thanks to the external environment (exogenous acquisition) through horizontal gene transfer from the environment or other bacteria, which can originate from microbial or human factors [2,21,135].

#### 4.5.1. Antimicrobial Resistance in Biofilm

Biofilm formation is triggered by several factors, including exposure of cells in the planktonic state to subinhibitory antibiotic concentrations [135]. Pathogens in the biofilm matrix are more resistant to antibiotics and host immune defenses, so a mature biofilm requires a higher concentration of an antimicrobial agent for its elimination than a bacterium in planktonic form. Few biofilm cells are exposed to the antibiotic, and the exopolysaccharide matrix reduces drug penetration into the colony by preventing it from reaching the deeper layers [136]. The effective concentration of some antibiotics against biofilm bacteria can be up to 100 or 1000 times higher than the concentration required to neutralize bacteria in the planktonic state [135].

Marcinkiewic et al. reported that biofilm-related antimicrobial resistance depends on several factors [135]. First, biofilm growth is associated with increased mutations, and biofilm formation genes are associated with antimicrobial resistance. The difference in bacterial density within the biofilm determines a gradient in nutrient and oxygen availability that affects the mode of action of some antibiotics [135]. For example, oxygen may be consumed entirely in the first layers of the biofilm, while anaerobic niches form in the deeper layers. The absence of oxygen prevents some antibiotics from penetrating the bacterial cells and exerting their bactericidal effect, converting them into a bacteriostatic effect.

Various nutrients may become scarce or fluctuate in concentration, altering bacterial metabolism and, consequently, the pH and CO_2_ concentration of the microenvironment or the vegetative state of the cells themselves. For example, a change in pH reduces the activity of aminoglycosides, while a nutrient deficiency can cause a state of cell non-growth, leading to the failure of β-lactam drugs that require actively growing cells to be effective [137]. Finally, increased efflux pump activity and activation of quorum sensing systems reduce and neutralize the antimicrobial agent attempting to penetrate [135].

#### 4.5.2. Antimicrobial Resistance in Major Periodontal Pathogens

Recent studies have investigated the emergence of antimicrobial resistance in some of the major pathogenic species of the oral cavity, including *Porphyromonas gingivalis*, *Aggregatibacter actinomycetemcomitans*, *Tannerella forsythia*, *Prevotella* spp., *Streptococcus* spp., *Staphylococcus aureus*, and *Candida* spp. [87,91,93].

At the oral level, the difficulty in eradicating periodontal pathogens means the progression of periodontitis with increased destruction of tooth-supporting tissues and loss of alveolar bone and teeth, eventually leading to masticatory dysfunction and the need for complex dental prosthetic rehabilitation [138,139,140]. These events can cause severe effects on the individual, both from a nutritional point of view (due to tooth loss, chewing ability is reduced, making the diet more restricted and unbalanced) and from a psychological point of view (tooth loss and the use of dentures can negatively impact the patient’s self-esteem), as well as from a systemic health point of view [141,142,143]. In addition, the difficulty of eradication may require more invasive procedures to counteract disease progression, such as opening a surgical flap or using bone and mucosal grafts [144].

Failure to eradicate antibiotic-resistant dental and periodontal pathogenic species results in an inflammatory condition in the oral cavity that can have systemic consequences [145]. Chronic periodontitis promotes the release of protein C-reactive protein, interleukin-1b and interleukin-6, and Tumor Necrosis Factor-alpha by neutrophils, which stimulate bone resorption and consequent destruction of periodontal tissue [145]. These proinflammatory cytokines, along with toxins and products of bacterial metabolism that may be present in the blood, promote the spread of inflammation throughout the body [145].

In addition to a systemic inflammatory state, oral bacteria may be associated with specific diseases such as cardiovascular disease, lung infections, oral cancer, diabetes, and neurodegenerative diseases such as Alzheimer’s disease [146]. The spread of antibiotic-resistant microorganisms from the oral cavity can be detrimental to any of the above conditions, leading to possible hospitalization, the need for more expensive treatment not always available, worse contraindications, and even endangering the life of the subject himself [145].

#### 4.5.3. Chlorhexidine and Antimicrobial Resistance

Chlorhexidine is a potent antiseptic widely used in dentistry. It was synthesized in the early 1950s in the United Kingdom by Imperial Chemical Industries as a potential antimalarial and introduced into dentistry in the late 1960s [147].

Chlorhexidine, 0.2% mouthwash, is one of the most used mouthwashes in dentistry and by the general population to reduce the bacterial load in the oral cavity [106] to control or prevent oral infections [148,149,150]. Chlorhexidine exerts its bactericidal properties through an increase in cell membrane permeability, which causes lysis and loss of intracellular material [151].

Studies by Tribble et al. and Brookes et al. on the effects of chlorhexidine on the oral microbiome show a decrease in colonizing species [151,152,153]. However, this decrease appears to be more harmful than beneficial. Indeed, chlorhexidine rinses lead to a pH reduction in the oral cavity, which may promote demineralization of enamel, and to a decrease in *Veillonella* species, which help regulate blood pressure by reducing nitrates to nitrites (from which nitric oxide is then formed, acting at the level of blood vessel smooth muscle) [151].

However, exposure to subinhibitory concentrations of chlorhexidine promotes the development of resistance [89]. In the oral cavity, such low concentrations depend on a mechanism of chlorhexidine inhibition by organic substances in saliva and serum proteins. In addition, due to poor penetration into biofilm, a chlorhexidine concentration gradient exists between the surface and deeper layers, resulting in subinhibitory concentrations in the innermost layers [147].

Low chlorhexidine concentrations lead to increased antimicrobial resistance in Gram-negative and Gram-positive bacteria due to changes in their cell membrane structure and ion pump function [154].

Short-term but repeated exposures to 0.12% chlorhexidine initially allow the inactivation of oral bacteria but may cause rapid re-growth of biofilms later. Prolonged and repeated exposures lead to the development of pathogenic species such as *S. mutans* and *Porphyromonas* [151].

A study by Saleem et al. investigated the chlorhexidine resistance of bacteria isolated from the dental plaque of five healthy patients and found that the bacteria were resistant not only to the antiseptic but also to several antibiotics such as ampicillin, gentamicin, tetracycline, and kanamycin [155].

The review by Cieplik et al. highlighted how chlorhexidine rinses could induce the formation of “persisters” (cells with a specific phenotype that gives them lower susceptibility) in *Candida albicans* biofilms, leading to a higher risk of developing candidiasis, especially in immunocompromised patients [147,156].

General factors and those specifically related to dental problems and practice contributing to antimicrobial resistance and/or antibiotic unresponsiveness are synthesized in Table 6.

#### 4.5.4. Measures to Counteract Antimicrobial Resistance in Dentistry: Antibiotic Stewardship

Healthcare professionals play a critical role in maintaining the effectiveness of antibiotics [68]. In fact, improper prescribing of antibiotics is the main factor that promotes the emergence of resistant microorganisms. However, many factors influence a physician’s decision to prescribe an antibiotic, and they can lead to the neglect of good practice: from diagnostic uncertainty to knowledge gaps, patient demand, or insufficient time to ensure treatment adherence [5,6,132,157].

The term “stewardship” refers to the careful and responsible use of something relied upon for one’s health, such as natural resources [59]. To curb antimicrobial resistance, every physician must know how to use antibiotics by prescribing them appropriately and educating their patients and colleagues on the proper use of this basic but increasingly limited medical resource [158,159,160,161].

Responsible use of antibiotics in clinical practice is based on proper diagnosis and prescribing antibiotics only when indicated and is based on the following principles: Prescribing the most appropriate drug at the correct dose, by the proper route of administration, and for the most appropriate duration [5].

Among the measures introduced by the World Health Organization to promote the conscious use of antibiotics is the new classification of these drugs into three groups with the acronym AWaRe (Access, Watch, and Reserve) [162].

The first group, Access, includes all antibiotics that offer the best therapeutic benefit with the lowest potential for resistance.

The second group, Watch, includes the agents most susceptible to selective resistance.

The third group, Reserve, consists of all those antibiotics, such as meropenem, that should be used little, especially in those microorganisms that have developed multi-resistance [14].

In addition, the WHO experts recommend a mnemonic trick to help dentists and physicians to remember the components of good antimicrobial stewardship, using the acronym MIND ME: “M—Microbiology must guide therapy whenever possible; I—Indications should be evidence-based; N—Narrowest spectrum required; D—Dosage appropriate to the site and type of infection; M—Minimize the duration of therapy; E—Ensure monotherapy in most cases” [163].

It begins with a careful clinical evaluation by taking the patient’s history and performing an objective examination. It must then be determined if further diagnostic testing is needed to decide what therapy to offer the patient.

**Figure 3:**
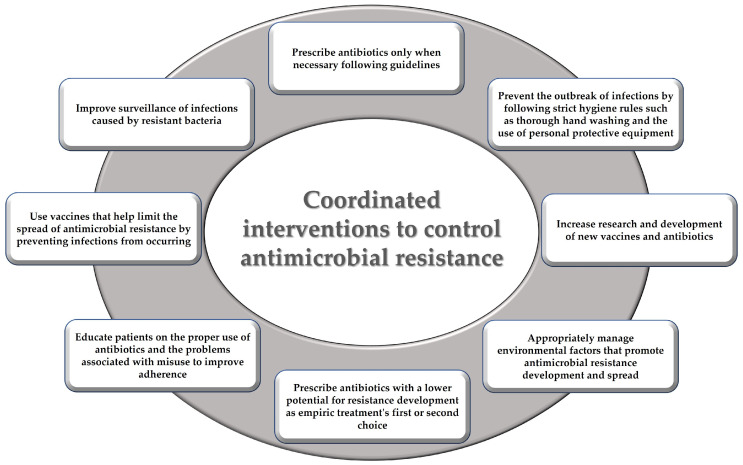
Coordinated interventions proposed to control antimicrobial resistance.

In this scenario, it has been recognized that it is necessary to control the phenomenon of antimicrobial resistance by promoting coordinated interventions in different areas (Figure 3) [164,165,166].

Like all narrative reviews, the present study is limited by the fact that it lacks a systematic and rigorous methodology for searching, selecting, and synthesizing evidence and is, therefore, subject to various biases, including publication, selection, and reviewer biases. Narrative reviews, however, can be useful in providing a broad overview of a topic and conveying knowledge in a simple and understandable way that is appropriate for clinicians. Indeed, the present review aimed at the discursive synthesis of current evidence to popularize the topic in a simple manner to readers who are not familiar with the topic itself, and increase their awareness of antimicrobial resistance in dentistry secondary to both overuse and misuse of antibiotics. This may be particularly important when addressing dentists’ attitudes toward antibiotic administration and awareness of antimicrobial resistance.

Retrieved evidence highlighted the need to establish more evidence-based and accurate antibiotic prescriptions in dentistry. Future perspectives should rely on sensitizing dentists and dental patients to minimize and rationalize the use of antibiotics only when it is indicated and necessary, enhancing knowledge and awareness of the antimicrobial resistance in dentistry, improving patients’ adherence to antimicrobial treatment, and developing alternative treatments, such as nutraceuticals, probiotics, or prebiotics. Moreover, the increasing knowledge of the oral microbiota brings us closer to personalized medicine and dentistry, based on individualized therapies and considering each person with its unique characteristics, and should be considered by current and future dentists to treat oral cavity infections better than before.

## 5. Conclusions

A total of 78 studies, 47 on the epidemiology of antibiotic use and prescription for therapy and prophylaxis in dentistry, 6 on antibiotic therapy in dentistry, 12 on antibiotic prophylaxis in dentistry, 0 on adherence of dental patients to antibiotic prescription, and 13 on antimicrobial resistance in dentistry, were presently considered.

A significant proportion of antibiotics prescribed for acute dental diseases and prophylaxis are unnecessary and inappropriate, leading to antibiotics overuse and misuse, increased bacterial resistance, and associated adverse outcomes. Antibiotics should only be prescribed in clinical situations requiring empiric antibiotic therapy and not for all odontogenic infections, and systemic antibiotics are recommended only for specific situations.

The two primary indications for antibiotic prophylaxis in dentistry are preventing bacterial endocarditis and surgical site infection.

Patient adherence to therapy is crucial for therapeutic success and for reducing the development of resistant bacterial strains.

Improper prescribing of antibiotics is the main factor that promotes the emergence of resistant microorganisms, and many factors can lead to neglect of good practice. Indeed, every physician must know how to properly use antibiotics and educate their patients and colleagues.

In conclusion, the responsible use of antibiotics in clinical practice is based on proper diagnosis and prescribing antibiotics only when indicated, and the World Health Organization recommends measures such as the new classification of antibiotics into three groups and the MIND ME acronym to promote the conscious use of antibiotics.

## Figures and Tables

**Table 1:** Characteristics of the included studies: first author, year and journal of publication, reference number, title, design, and synthesis of the main conclusion(s) about the epidemiology of antibiotic consumption and prescription in dentistry reported in the included studies.

Epidemiology of Antibiotic Consumption and Prescription in Dentistry		
Study Characteristics	Title	Conclusion(s)
Ahsan, 2020 *PLoS One* [17] Cross-sectional	“Antibiotic prescription patterns for treating dental infections in children among general and pediatric dentists in teaching institutions of Karachi, Pakistan”	Most dentists, especially with a high volume of pediatric patients, have not shown adherence to professional guidelines in prescribing antibiotics to treat dental infections in children.
Al-Harthi, 2013 *Saudi Med J* [18] Cross-sectional	“Appraisal of antimicrobial prescribing practices of governmental and non-governmental dentists for hospitals in the western region of Saudi Arabia”	Professional guidelines for prescribing antibiotics were not considered attractive to all respondents. The questionnaire showed divergence in antibiotic prescribing among different specialists.
Al-Harthi, 2015 *Saudi Med J* [19] Cross-sectional	“Perceptions and knowledge regarding antimicrobial stewardship among clinicians in Jeddah, Saudi Arabia”	The questionnaire showed divergence in antibiotic prescribing among different specialists, with greater adherence by primary care physicians than other specialists, including dentists. More knowledge was demonstrated on the topic of antimicrobial resistance.
Al-Johani, 2017 *Niger J Clin Pract* [20] Cross-sectional	“Pattern of prescription of antibiotics among dental practitioners in Jeddah, KSA: A cross-sectional survey”	The questionnaire showed a lack of adherence to antibiotic prescribing guidelines among dentists in Jeddah. The antibiotic most commonly used by dentists for most orofacial infections was amoxicillin (73.8%).
Al-Taani, 2022 *J Infect Dev Ctries* [21] Cross-sectional	“Antibiotic use and resistance: Information sources and application by dentists in Jordan”	The questionnaire showed that only 35.5% of dentists surveyed refer to published guidelines for proper antibiotic prescribing in dentistry. Only 9.3% of dentists were aware of national action plans on antibiotic resistance. More than half of the respondents expressed a desire to receive more information on the proper use of antibiotics.
Baskaradoss, 2018 *J Investing Clin Dent* [22] Cross-sectional	“Pattern of antibiotic prescription among dentists in Riyadh, Saudi Arabia”	The most frequently prescribed antibiotic was amoxicillin. Most dentists (more than 70%) prescribed antibiotics to heart patients. Dentists with higher educational qualifications followed more appropriate prescribing patterns than their other colleagues. Antibiotic prescribing patterns were inappropriate among Riyadh dentists.
Bhuvaraghan, 2021 *Antibiotics (Basel)* [23] Systematic Review	“Antibiotic Use and Misuse in Dentistry in India-A Systematic Review”	The study showed significant abuse or misuse in prescribing antibiotics for prophylactic and therapeutic purposes in dentistry, particularly for the use of broad-spectrum antibiotics. Antibiotic self-medication for dental problems by the general population was found to be widespread.
Bird, 2018 *Br Dent J* [24] Cross-sectional	“Higher antibiotic prescribing propensity of dentists in deprived areas and those with greater access to care in the North East and Cumbria”	The rate of antibiotic prescriptions in dentistry is recorded to be higher in deprived areas of the North East and Cumbria. Areas with similar deprivation have shown that the prescribing rate depends on the bias or preferences of the practitioner.
Cope, 2016 *Br J Gen Pract* [25] Retrospective	“Dental consultations in UK general practice and antibiotic prescribing rates: A retrospective cohort study”	In the United Kingdom, consultation rates for dental reasons in general practice are low, but more than half hesitate to prescribe antibiotics. This raises concerns about antibiotic resistance and patient morbidity.
Cope, 2016 *Community Dent Oral Epidemiol* [26] Cross-sectional	“Antibiotic prescribing in UK general dental practice: A cross-sectional study”	The study showed a high rate of inappropriate antibiotic prescriptions among general dental practitioners. In the healthcare setting, numerous antibiotic prescriptions in the absence of infection haves been associated with clinical temporal pressures and patient characteristics, such as expectations for treatment with antibiotics and refusal of surgical treatment.
D’Ambrosio, 2022 *Healthcare (Basel)* [5] Cross-sectional	“Attitudes towards Antibiotic Prescription and Antimicrobial Resistance Awareness among Italian Dentists: What Are the Milestones?”	The main reasons for prescribing antibiotics in dentistry were abscesses, extractions, and pulpits. In case of allergy to penicillins, most dentists have prescribed macrolides, but only a small fraction of them have consulted the guidelines for an antibiotic prescription. In Italy, a high prevalence of misuse and overuse of antibiotics was recorded, similar to other countries.
Duncan, 2021 *Br Dent J* [27] Cross-sectional	““You had to do something”: prescribing antibiotics in Scotland during the COVID-19 pandemic restrictions and remobilization”	Antibiotic prescriptions increased by 49% after the suspension of routine dental care due to the COVID-19 pandemic. The data showed that following remobilization, antibiotic prescribing remained about 28% higher than in the pre-pandemic period. The survey showed that dentists were concerned about the increased use of antibiotics.
Durkin, 2019 *J Am Dent Assoc* [28] Cross-sectional	“Knowledge and attitudes of recently qualified dentists working in Wales towards antimicrobial prescribing and resistance”	The study found that recently qualified dentists working in Wales were influenced by the use of guidelines and teachings received from students for antibiotic prescriptions. However, most were not confident in treating acute dental conditions. Antibiotic prescribing was also influenced by pressures induced by patients.
Epstein, 2000 *J Am Dent Assoc* [29] Cross-sectional	“A survey of antibiotic use in dentistry”	The questionnaire showed that about 85% of respondents appropriately prescribed antibiotics in dentistry for therapeutic use for dosage and duration. More than 80% of dentists said they follow the American Heart Association’s guidelines for antibiotic prophylaxis, which has been prescribed more frequently for patients with a history of rheumatoid fever, joint replacements, and heart murmur.
Farkaš, 2021 *Microb Drug Resist* [30] Cross-sectional	“Antibiotic Prescribing Habits and Antimicrobial Resistance Awareness of Dental Practitioners in Primorsko-Goranska County, Croatia”	The study showed the overuse of antibiotics among dentists in Croatia, particularly in cases where surgical treatment was the indication of first choice. Broad-spectrum antibiotics were the most prescribed drugs.
Garg, 2014 *J Antimicrob Chemother* [31] Cross-sectional	“Antibiotic prescription pattern among Indian oral healthcare providers: A cross-sectional survey”	The study showed that antibiotics were overprescribed in India, particularly in irreversible pulpitis, necrotic pulp, and acute apical periodontitis. Amoxicillin, with or without clavulanic acid, was the antibiotic of first choice by dentists for nonallergic patients.
George, 2022 *J Pharm Bioallied Sci* [32] Cross-sectional	“Antimicrobial prescription patterns among oral implantologists of Kerala, India: A cross-sectional survey”	Most systemic antibiotic prescriptions in implant surgery were not in accordance with current evidence. Many implantologists prescribed systemic antibiotics for the prevention of infection following simple implant insertions.
Gowri, 2015 *J Orofacial Sci* [33] Cross-sectional	“Antibiotic use in dentistry: A cross-sectional survey from a developing country”	The study showed the lack of knowledge, attitude, and practice of antibiotic use among dentists at a university and hospital institution.
Goulao, 2021 *Implement Sci* [34] Randomised Control Trial	“Audit and feedback with or without training in-practice targeting antibiotic prescribing (TiPTAP): a study protocol of a cluster randomised trial in dental primary care”	Training courses to improve the appropriateness of antibiotic prescriptions in primary dental care have shown good results.
Jones, 2018 *Eur J Dent Educ* [35] Cross-sectional	“Knowledge and attitudes of recently qualified dentists working in Wales towards antimicrobial prescribing and resistance”	Recently qualified dentists in Wales reported that the guidelines and teachings received in the course of the study were the main factors influencing their choices for prescribing antibiotics. However, some participants still did not feel confident in prescribing antibiotics for some acute dental conditions.
Kusumoto, 2021 *BMC Oral Health* [36] Retrospective	“Effect of educational intervention on the appropriate use of oral antimicrobials in oral and maxillofacial surgery: a retrospective secondary data analysis”	The study showed that educational intervention helped dentists prescribe antibiotics more appropriately than in the past.
Licata, 2021 *Antimicrob Agents Chemother* [37] Cross-sectional	“Endodontic Infections and the Extent of Antibiotic Overprescription among Italian Dental Practitioners”	Acute abscesses without systemic involvement and acute apical periodontitis were most frequently associated with antibiotic overprescription among Italian dentists.
Lokhasudhan, 2017 *J Adv Pharm Educ Res* [38] Cross-sectional	“Knowledge, attitude, and practice survey on usage of antibiotics among dental practitioners in southern region of India”	The study showed the over-prescription of antibiotics as an intracanal medicine for prophylaxis before endodontic treatment and for the management of patients with systemic diseases among endodontists and neoendodontists.
Mansour, 2018 *Pharm Pract (Granada)* [39] Cross-sectional	“Knowledge, practice and attitudes regarding antibiotics use among Lebanese dentists”	A lack of uniformity with the guidelines was found in the prophylactic and therapeutic prescriptions of Lebanese dentists. The latter showed greater knowledge of the problems related to antibiotic resistance.
McKay, 2020 *Br Dent J* [40] Cross-sectional	“An analysis of the clinical appropriateness of out-of-hours emergency dental prescribing of antibiotics in Northern Ireland”	The study reported that a high number of antibiotic prescriptions made in out-of-hours emergency dental clinics in Northern Ireland did not follow guidelines.
Mengari, 2020 *JRMDS* [41] Cross-sectional	“Knowledge and Practice of Antibiotic Prescription Among Dentists for Endodontic Emergencies”	The study showed that antibiotic prescriptions for endodontic emergencies were different among dentists working in governmental or private sectors or in educational institutions. General dentists prescribed antibiotics more frequently, even when it was not necessary, than endodontists.
Mustafa, 2022 *Eur J Dent* [42] Cross-sectional	“Administration of Systemic Antibiotics for Dental Treatment in Kosovo Major Dental Clinics: A National Survey”	Prescriptions of amoxicillin with or without clavulanic acid increased dramatically from 2015 to 2019 in dental clinics in Kosovo.
Nourah, 2021 *Int J Med Dent* [43] Cross-sectional	“Prescribing practice of systemic antibiotics by periodontists in Saudi Arabia”	Patterns of systemic antibiotic prescriptions were heterogeneous among periodontists in Saudi Arabia.
Ogunbodede, 2005 *J Contemp Dent Pract* [44] Retrospective	“Retrospective survey of antibiotic prescriptions in dentistry”	The total number of drugs prescribed for dental reasons ranged from one to seven, and penicillins were the most prescribed antibiotic. The dose, frequency, and duration were wrong in some prescriptions. Indications on the best time to take the antibiotic in relation to meals had not been specified in any prescription.
Ono, 2020 *PLoS One* [45] Cross-sectional	“The first national survey of antimicrobial use among dentists in Japan from 2015 to 2017 based on the national database of health insurance claims and specific health checkups of Japan”	Cephalosporins were the antibiotic most prescribed by Japanese dentists between 2015 and 2017. At the same time, around 99% of outpatients had been prescribed an antibiotic.
Osailan, 2021 *J Pharm Policy Pract* [46] Cross-sectional	“Knowledge and Attitude towards Antibiotics Prescription and Antimicrobial Resistance among Dental Surgeons in Saudi Arabia”	The study showed that antibiotic prescriptions among Saudi Arabian oral surgeons were inappropriate in most cases. Young age, male gender, higher level of studies, and poor aptitude were factors related to inappropriate antibiotic use.
Palmer, 2001 *J Antimicrob Chemother* [47] Cross-sectional	“Antibiotic prescribing knowledge of National Health Service general dental practitioners in England and Scotland”	The study showed that dentists who had taken at least one postgraduate course on antibiotic use had significantly greater knowledge. Significant differences were also found in the appropriateness of antibiotic prescriptions of dentists in English Health Authorities compared to dentists in Scottish Health Boards.
Palmer, 2019 *Prim Dent J* [48] Cross-sectional	“A Pilot Study to Investigate Antibiotic Prescribing in Private Dental Practice in the UK”	The study showed that UK dentists working in private structures prescribed antibiotics less and more appropriately than NHS dentists.
Pisarnturakit, 2020 *Int J Health Plann Manage* [49] Cross-sectional	“Managing knowledge for health care quality: An investigation of rational antibiotic use among Thai dentists”	Thai dentists have been largely shown to use antibiotics appropriately. Mobile applications were reported as the preferred means of filling the remaining gaps in knowledge of antibiotic use.
Rodriguez-Núñez, 2009 *J Endod* [50] Cross-sectional	“Antibiotic Use by Members of the Spanish Endodontic Society”	Most members of the Spanish Endodontic Society had indicated the appropriate antibiotic therapy for several orofacial infections. However, some inappropriate choices were made in some cases of minimal or no infections.
Rubanenko, 2021 *Antibiotics (Basel)* [51] Cross-sectional	“Assessment of the knowledge and approach of general dentists who treat children and pediatric dentists regarding the proper use of antibiotics for children”	The level of knowledge about when antibiotics should or should not be used in children is poor among both general and pediatric dentists. Antibiotic prescriptions, in a few cases, have been in accordance with European and American Pediatric Dentistry guidelines.
Salako, 2004 *J Dent* [52] Cross-sectional	“Pattern of antibiotic prescription in the management of oral diseases among dentists in Kuwait”	The study showed a lack of uniformity in prescribing antibiotics for oral diseases among Kuwaiti dentists. Uncertainty in diagnosis, patient expectations, and lack of time for immediate treatment were the main factors influencing antibiotic prescriptions.
Schmidt, 2021 *Int J Environ Res Public Health* [53] Systematic Review	“A Review of Evidence-Based Recommendations for Pericoronitis Management and a Systematic Review of Antibiotic Prescribing for Pericoronitis among Dentists: Inappropriate Pericoronitis Treatment Is a Critical Factor of Antibiotic Overuse in Dentistry”	Pericoronitis was the second leading cause of antibiotic use in dentistry. Antibiotics, particularly amoxicillin or metronidazole, were given to more than half of the subjects with pericoronitis. Antibiotic prescriptions were inappropriate and noncompliant with guidelines in most cases.
Sefah, 2022 *JAC-antimicrobial resistance* [54] Retrospective	“Evaluation of antibiotic prescribing for ambulatory patients seeking primary dental care services in a public hospital in Ghana: a clinical audit study”	Antibiotics were prescribed to more than 90 percent of patients who required primary dental care services in Ghana’s public hospitals. In almost all cases, the antibiotic prescription was not in accordance with the Ghana Standard Treatment Guidelines.
Segura-Egea, 2010 *Int Endod J* [55] Cross-sectional	“Pattern of antibiotic prescription in the management of endodontic infections amongst Spanish oral surgeons”	Most members of the Spanish Oral Surgery Society had selected the appropriate antibiotic for endodontic infections; however, still, many prescribed the antibiotics inappropriately. Odontologists have more frequently prescribed antibiotics than stomatologists for necrotic teeth with sinus tract and chronic apical periodontitis.
Shalini, 2022 *Indian Drugs* [56] Cross-sectional	“Knowledge and attitude of antibiotic prescription among implantologists: an observational study”	The study showed a lack of congruence among implantologists about the recommended protocols for the use of antibiotics for the prophylaxis or management of implant complications.
Shemesh, 2022 *Clin Oral Investig* [57] Cross-sectional	“International questionnaire study on systemic antibiotics in endodontics. Part 1. Prescribing practices for endodontic diagnoses and clinical scenarios”	The study showed a lack of congruence between the recommended protocols for the use of systemic antibiotics for endodontic treatment and the clinical prescriptions of Israeli and Soviet dentists.
Sneddon, 2022 *Antibiotics* [58] Cross-sectional	“Exploring the Use of Antibiotics for Dental Patients in a Middle-Income Country: Interviews with Clinicians in Two Ghanaian Hospitals”	The rate of antibiotic prescriptions in dentistry had been influenced by the work environment, clinical issues such as lack of available sterile instrumentation, and patient preferences or needs.
Teoh, 2020 *Antibiotics (Basel)* [59] Systematic Review	“Measuring Antibiotic Stewardship Programmes and Initiatives: An Umbrella Review in Primary Care Medicine and a Systematic Review of Dentistry”	Most of the antibiotic prescriptions (80%) take place in dentistry, but the increase in prescriptions also includes primary medical care.
Teoh, 2019 *BMC Oral Health* [60] Cross-sectional	“A survey of prescribing practices by general dentists in Australia”	Dentists with less than five years since graduation had a lower rate of antibiotic prescription abuse than their colleagues. Years of practice were the demographic factor that most influenced the antibiotic prescription rate.
Thompson, 2019 *J Antimicrob Chemother* [61] Systematic Review	“Factors associated with antibiotic prescribing for adults with acute conditions: an umbrella review across primary care and a systematic review focusing on primary dental care”	Factors potentially influencing antibiotic prescriptions were clinician-related, clinical context-related, patient-related, and social–political context-related.
Verma, 2022 *World J Dentistry* [62] Cross-sectional	“Antibiotic Prescribing Practices amongst the Dental Practitioners of Bhubaneswar City: A Cross-sectional Study”	The study showed gaps in knowledge of antibiotic prescription guidelines among dentists in the city of Bhubaneswar.

**Table 2:** Characteristics of the included studies: first author, year and journal of publication, reference number, title, design, and synthesis of the main conclusion(s) about antibiotic therapy in dentistry reported in the included studies.

Antibiotic Therapy in Dentistry		
Study Characteristics	Title	Conclusion(s)
Cope, 2015 *BMJ Open* [63] Qualitative Study	“General practitioners’ attitudes towards the management of dental conditions and use of antibiotics in these consultations: a qualitative study”	Antibiotics were considered the first-line treatment for many primary care physicians for the immediate management of acute dental problems. Often the patients themselves required the administration of the antibiotic. General practitioners who prescribed antibiotics rarely wanted to encourage patients to visit dentistry.
Froum, 2015 *Int J Periodontics Restorative Dent* [64] Cross-sectional	“An evaluation of antibiotic use in periodontal and implant practices”	The prescription, duration, and initiation of antibiotics were very heterogeneous in the ten therapeutic treatments in periodontal and implant practice, such as the treatment of acute and chronic periodontitis, sinus lifts or crest augmentation surgery, and immediate or delayed implant placement.
Kaul, 2021 *J Family Med Prim Care* [65] Cross-sectional	“Oral pain and infection control strategies for treating children and adolescents in India”	The study recorded the overuse of antibiotics and analgesics, especially in the postoperative phase and by general dentists, compared to specialists, for pain control and treatment of infections in children and adolescents.
Khattri, 2020 *Cochrane Database Syst Rev* [66] Systematic Review with Meta-analysis	“Adjunctive systemic antimicrobials for the non-surgical treatment of periodontitis”	No statistically significant evidence was found on the long-term efficacy of systemic antibiotics used in addition to non-surgical periodontitis treatments. The superiority of one antibiotic over the others has not been defined either.
Silva, 2017 *SPMED* [67] Cross-sectional	“The use of systemic antibiotics in endodontics: a cross-sectional study”	A considerable part of Portuguese dentists had inappropriately prescribed antibiotics as a therapeutic treatment for inflammatory endodontic diseases.
Thompson, 2022 *Trials* [68] Cross-sectional	“Dental antibiotic stewardship: study protocol for developing international consensus on a core outcome set”	The study showed that current international guidelines for antibiotic therapy in dentistry are very heterogeneous.

**Table 3:** Characteristics of the included studies: first author, year and journal of publication, reference number, title, and design. Synthesis of the main conclusion(s) about antibiotic prophylaxis in dentistry reported in the included studies.

Antibiotic Prophylaxis in Dentistry		
Study Characteristics	Title	Conclusion(s)
Bianco, 2021 *Antibiotics (Basel)* [69] Cross-sectional	“Appropriateness of Antibiotic Prescription for Prophylactic Purposes among Italian Dental Practitioners: Results from a Cross-Sectional Study”	The prescription of antibiotics by Italian dentists for prophylactic reasons was found to be not in accordance with the guidelines in 70.9% of cases.
Ireland, 2012 *Br Dent J* [70] Cross-sectional	“An investigation of antibiotic prophylaxis in implant practice in the UK”	Pre- and post-operative prescription regimens of antibiotics in dental implant practice in the United Kingdom are highly variable. In most cases, antibiotics were prescribed to prevent infection of the surgical site or to reduce bacteremia.
Kirnbauer, 2022 *Clin Oral Investig* [71] Randomized Control Trial	“Is perioperative antibiotic prophylaxis in the case of routine surgical removal of the third molar still justified? A randomized, double-blind, placebo-controlled clinical trial with a split-mouth design”	Oral antibiotics administered in the perioperative phase of routine surgical extractions of wisdom teeth in the absence of inflammation did not show a reduction in post-operative complications or greater benefit in patient-related outcome measures.
Lollobrigida, 2021 *Antibiotics (Basel)* [72] Cross-sectional	“Antibiotics to Prevent Surgical Site Infection (SSI) in Oral Surgery: Survey among Italian Dentists”	The choices reported by the dentists about antibiotic prescriptions for the prevention of infections in post-extraction surgical sites were appropriate for deciduous tooth extractions and simple extractions in healthy adult subjects. However, responses were more heterogeneous in adult extractions with comorbidities, complex or multiple extractions, drainage abscesses, and implant placement. The dosage to be used was also found to be very heterogeneous.
Salgado-Peralvo, 2021 *Antibiotics (Basel)* [73] Systematic Review	“Preventive Antibiotic Therapy in the Placement of Immediate Implants: A Systematic Review”	The study showed the efficacy of prophylactic treatment with 2–3 g of amoxicillin one hour before immediate implant placement and after for 5–7 days at a dosage of 500 mg every 8 h to reduce the rate of early failure. In subjects with penicillin allergy, azithromycin, clarithromycin, or metronidazole were recommended, but not clindamycin, if possible.
Salgado-Peralvo, 2022 *Antibiotics (Basel)* [74] Systematic Review	“Is Antibiotic Prophylaxis Necessary before Dental Implant Procedures in Patients with Orthopaedic Prostheses? A Systematic Review”	There is no evidence showing a relationship between implant placement and an increased risk of orthopedic prosthesis infection. Therefore, the authors concluded that antibiotic prophylaxis is not justified in these cases.
Salgado-Peralvo, 2022 *J Stomatol Oral Maxillofac Surg* [75] Systematic Review	“Preventive antibiotic therapy in bone augmentation procedures in oral implantology: A systematic review”	The study showed that the administration of 2/3 g of amoxicillin one hour before bone augmentation procedures in oral implantology allowed a reduction in the rate of early implant failure and the infection risk of the grafted bone particles.
Sato, 2022 *Oral Dis* [76] Retrospective	“Amoxicillin vs. third-generation cephalosporin for infection prophylaxis after third molar extraction”	Antibiotic prophylaxis with amoxicillin prior to extraction of impacted third molars showed a lower incidence of surgical site infections than using third-generation cephalosporins.
Sato, 2022 *J Infect Chemother* [77] Retrospective	“Trends in prophylactic antibiotic use for tooth extraction from 2015 to 2018 in Japan: An analysis using a health insurance claims database”	The trend in antibiotic prophylaxis for the extraction of third molars changed from 2015 to 2018 in Japan after the National Action Plan, with an increase in the use of amoxicillin and a decrease in third-generation cephalosporins.
Sologova, 2022 Dent J (Basel) [78] Systematic Review	“Antibiotics Efficiency in the Infection Complications Prevention after Third Molar Extraction: A Systematic Review”	The study showed that amoxicillin, with or without clavulanic acid, is the most widely used antibiotic at different dosages and durations to prevent infectious complications following the extraction of third molars.
Sing Gill, 2018 *Medicina (Kaunas)* [79] Systematic Review with Meta-Analysis	“A Systematic Review and Meta-Analysis Evaluating Antibiotic Prophylaxis in Dental Implants and Extraction Procedures”	The use of antibiotics prophylactically for third-molar extractions in healthy patients was supported by little evidence. In contrast, no significant evidence showed a higher incidence of adverse events to antibiotics compared with placebo.
Williams, 2020 *Br Dent J* [80] Cross-sectional	“Antibiotic prophylaxis during dental implant placement in the UK”	The study showed heterogeneous choices to prescribe antibiotics prophylactically for implant placement. Almost half of the dentists did not prescribe antibiotics routinely. In other cases, antibiotics were prescribed for complex procedures and by more qualified dentists.

**Table 4:** Characteristics of the included studies: first author, year and journal of publication, reference number, title, and design. Synthesis of the main conclusion(s) about dental patients’ adherence to antibiotic prescription reported in the included studies.

Dental Patients’ Adherence to Antibiotic Prescription	
Total	0 studies

**Table 5:** Characteristics of the included studies: first author, year and journal of publication, reference number, title, and design. Synthesis of the main conclusion(s) about antimicrobial resistance in dentistry reported in the included studies.

Antimicrobial Resistance in Dentistry		
Study Characteristics	Title	Conclusion(s)
Abe, 2022 *Front Microbiol* [81] Systematic Review with Meta-Analysis	“Antimicrobial resistance of microorganisms present in periodontal diseases: A systematic review and meta-analysis”	No evidence was found for the presence of specific antibiotic resistance profiles in microorganisms implicated in periodontal disease. The highest antibiotic resistance recorded was for ampicillin, while the lowest was for ciprofloxacin.
Abe, 2018 *Medicine* [82] Systematic Review	“Resistance profile to antimicrobial agents in the main circulating bacteria isolated from acute periodontal and endodontic infections in Latin America (MICROBE- DENT) A systematic review protocol”	Further studies are needed to assess the prevalence of antimicrobial resistance in endodontics and periodontics in Latin America.
Almeida, 2020 *PLoS One* [83] Cross-sectional	“Bacterial diversity and prevalence of antibiotic resistance genes in the oral microbiome”	No significant differences were found between the taxonomies of healthy or diseased oral microbiomes. However, healthy subjects showed a more diverse microbiological community. At least one antibiotic resistance gene was found in 72.7% of the samples.
Alzahrani, 2020 *Risk Manag Healthc Policy* [84] Retrospective	“Inappropriate Dental Antibiotic Prescriptions: Potential Driver of the Antimicrobial Resistance in Albaha Region, Saudi Arabia”	Misuse and abuse of antibiotic prescriptions have been found among dentists in Saudi Arabia. The inappropriate use of antibiotics could lead to the development of antibiotic resistance phenomena.
Groppo, 2005 *Gen Dent* [85] Clinical Trial	“Antimicrobial resistance of Staphylococcus aureus and oral streptococci strains from high-risk endocarditis patients”	Microorganisms causing bacterial endocarditis have shown high rates of antimicrobial resistance to antibiotics commonly used for prophylaxis in dentistry.
Irshad, 2020 *Antibiotics (Basel)* [86] Clinical Trial	“Characterization and Antimicrobial Susceptibility of Pathogens Associated with Periodontal Abscess”	The study showed that several bacterial species isolated from periodontal abscesses had high rates of antimicrobial resistance to amoxicillin, tetracyclines, and metronidazole, while azithromycin was not associated with antimicrobial resistance in these cases.
Kiros, 2022 *Biomed Res Int* [87] Cross-sectional	“Bacterial Profile, Antimicrobial Susceptibility Pattern, and Associated Factors among Dental Caries-Suspected Patients Attending the Ayder Comprehensive Specialized Hospital and Private Dental Clinic in Mekelle, Northern Ethiopia”	The study recorded multidrug resistance of 40.4% of microorganisms associated with dental caries. The highest resistance rate was found for penicillin and tetracyclines.
Lang, 2016 *Int J Antimicrob Agents* [88] Systematic Review with Meta-Analysis	“Resistance profiles to antimicrobial agents in bacteria isolated from acute endodontic infections: systematic review and meta-analysis”	The study showed that the antibiotic resistance profiles of bacteria causing acute endodontic infections were lower for amoxicillin. In addition, resistance rates increased when multiple cycles of antibiotics were given.
Laumen, 2021 *Front Microbiol* [89] Clinical Trial	“Sub-Inhibitory Concentrations of Chlorhexidine Induce Resistance to Chlorhexidine and Decrease Antibiotic Susceptibility in Neisseria gonorrhoeae”	The frequent use of chlorhexidine led to an increased rate of resistance of Neisseria gonorrhoeae to chlorhexidine itself and other antibiotics.
Rodrigues, 2004 *J Clin Periodontol* [90] Clinical Trial	“Antibiotic resistance profile of the subgingival microbiota following systemic or local tetracycline therapy”	Local or systemic administration of tetracycline in subjects with chronic periodontitis resulted in the transient selection of subgingival microorganisms intrinsically resistant to tetracycline itself.
Santos, 2002 *Anaerobe* [91] Clinical Trial	“Susceptibility of Prevotella intermedia/Prevotella nigrescens (and Porphyromonas gingivalis) to propolis (bee glue) and other antimicrobial agents”	Prevotella intermedia and Prevotella nigrescens were susceptible to penicillins, meropenem, erythromycin, and metronidazole, while a smaller percentage were susceptible to tetracyclines and a considerable number were resistant to clindamycin. Propolis was an effective alternative to periodontal pathogens.
van Winkelhoff, 2000 *J Clin Periodontol* [92] Comparative Study	“Antimicrobial resistance in the subgingival microflora in patients with adult periodontitis. A comparison between The Netherlands and Spain”	The increased use of antibiotics in Spain has led to a higher rate of antibiotic resistance of subgingival microorganisms in adult subjects with periodontitis. The resistance rate was found to be much lower among Dutch patients.
Vijayashree, 2018 *J Clin Diagnostic Res* [93] Retrospective	“Enterococcus faecalis an Emerging Microbial Menace in Dentistry-An Insight into the In silico Detection of Drug Resistant Genes and Its Protein Diversity”	Enterococcus Faecalis has been associated with endodontic and periodontal infections. The study showed that the bacterial genome hosts one or more genes encoding resistance to the most common antibiotics used in dentistry.

**Table 6:** Factors contributing to antimicrobial resistance/antibiotic unresponsiveness.

General Factors	Mechanisms
Food chain	Vegetables and meats from crops and farms using massive doses of antibiotics lead to the selection of super-resistant bacteria that can be transmitted to the final consumer (the human) when not adequately sanitized
Iatrogenic	Over-prescriptions; Broad-spectrum prescriptions; Wrong diagnosis (viral infections and other non-bacterial diseases mistreated with antibiotics); Patient’s empiric self-medications (wrong diagnosis, wrong molecules, and wrong posology).
Additional factors in dentistry	
Bacterial plaque and biofilm	A mature and thick dental plaque creates a progressive lower penetration of the antibiotic molecules through the biofilm layers, from the outer to the inner ones, thus invalidating the effectiveness of topical antibiotics and making systemic ones useless
Local inflammation	Local inflammation, as those occurring in periodontitis, is responsible for changes in pH that affect the antibiotic activities
Chlorhexidine	A pH reduction in the oral cavity, which may promote demineralization of enamel, and a decrease in *Veillonella* species, which help regulate blood pressure by reducing nitrates to nitrites; Subinhibitory concentrations of chlorhexidine promote the development of resistance, mainly at the inner layers of a thick biofilm; Prolonged and repeated exposures to 0.12% chlorhexidine lead to the development of pathogenic species such as *S. mutans* and *Porphyromonas;* Bacteria resistant to chlorhexidine also show resistance to several antibiotics, such as ampicillin, gentamicin, tetracycline, and kanamycin; Chlorhexidine rinses could induce the selection of *Candida albicans* strains less responsive to antiseptics, thus leading to a higher risk of developing candidiasis, especially in immunocompromised patients.

## Data Availability

Data are freely available on PubMed/MEDLINE, Scopus, and Google Scholar databases.
